# EMG-Based 3D Hand Motor Intention Prediction for Information Transfer from Human to Robot

**DOI:** 10.3390/s21041316

**Published:** 2021-02-12

**Authors:** Aberham Genetu Feleke, Luzheng Bi, Weijie Fei

**Affiliations:** School of Mechanical Engineering, Beijing Institute of Technology, Beijing 100081, China; 7520190125@bit.edu.cn (A.G.F.); 3120170241@bit.edu.cn (W.F.)

**Keywords:** Electromyography (EMG), 3-D movement, continuous motion, motor intention, hand motion

## Abstract

(1) Background: Three-dimensional (3-D) hand position is one of the kinematic parameters that can be inferred from Electromyography (EMG) signals. The inferred parameter is used as a communication channel in human–robot collaboration applications. Although its application from the perspective of rehabilitation and assistive technologies are widely studied, there are few papers on its application involving healthy subjects such as intelligent manufacturing and skill transfer. In this regard, for tasks associated with complex hand trajectories without the consideration of the degree of freedom (DOF), the prediction of 3-D hand position from EMG signal alone has not been addressed. (2) Objective: The primary aim of this study is to propose a model to predict human motor intention that can be used as information from human to robot. Therefore, the prediction of a 3-D hand position directly from the EMG signal for complex trajectories of hand movement, without the direct consideration of joint movements, is studied. In addition, the effects of slow and fast motions on the accuracy of the prediction model are analyzed. (3) Methods: This study used the EMG signal that is collected from the upper limb of healthy subjects, and the position signal of the hand while the subjects manipulate complex trajectories. We considered and analyzed two types of tasks with complex trajectories, each with quick and slow motions. A recurrent fuzzy neural network (RFNN) model was constructed to predict the 3-D position of the hand from the features of EMG signals alone. We used the Pearson correlation coefficient (CC) and normalized root mean square error (NRMSE) as performance metrics. (4) Results: We found that 3-D hand positions of the complex movement can be predicted with the mean performance of CC = 0.85 and NRMSE = 0.105. The 3-D hand position can be predicted well within a future time of 250 ms, from the EMG signal alone. Even though tasks performed under quick motion had a better prediction performance; the statistical difference in the accuracy of prediction between quick and slow motion was insignificant. Concerning the prediction model, we found that RFNN has a good performance in decoding for the time-varying system. (5) Conclusions: In this paper, irrespective of the speed of the motion, the 3-D hand position is predicted from the EMG signal alone. The proposed approach can be used in human–robot collaboration applications to enhance the natural interaction between a human and a robot.

## 1. Introduction

Currently, several applications, such as teleoperation [1], intelligent vehicles and aircraft [2], assistive and rehabilitation technology [3], and robot-assisted surgery [4] require the collaboration of human and robot partners to accomplish a shared task. To implement efficient communication in a human–robot collaboration system, both humans and robots need to understand the current state of their partner and be able to predict what they will do next [4,5]. Robots are enabled to infer human intentions in human–robot collaborative tasks through various modes, such as biological signals [6,7,8,9]. Biological signals can infer movement intention for intuitive and natural interaction between humans and robots. Electromyogram (EMG), which is one of these signals, is widely used for the estimation of simultaneous and proportional motor intention [10,11,12,13].

In robotic applications, the EMG signal is used as a communication channel between a human and a robot by inferring human intention that is transferred to robot control. Specifically, it is used to infer the motion parameters of the upper limb, which are the most active parts of the human body. In this regard, the simultaneous and proportional motion prediction from EMG signals and its application are currently a hot research topic. The predicted parameters of upper limb motions can be kinetic or kinematic, such as joint angles or hand positions. Since the relationship between the EMG signal and kinetic parameters is more direct than that of kinematic parameters, the estimation of kinetic parameters has been widely studied in the context of myoelectric control [13]. Therefore, in this paper, the mapping of the EMG signal to kinematic parameters, which is a 3-D hand position, is studied.

The simultaneous and proportional motion prediction for multiple DOFs at a single joint or multiple joints of upper limb motions have been studied by several researchers. Muceli et al. [14] and Bao et al. [15] proposed models to infer joint angle for the simultaneous activation of the multiple DOFs of the wrist. These studies are focused on the simultaneous and proportional motion prediction for multiple DOFs at a single joint. However, Pan et al. [16], Zhang et al. [17], and Liu et al. [18] proposed methods to simultaneously predict multiple DOFs across multiple joints such as shoulder, elbow, and wrist. The methods that are used for the simultaneous and proportional motion prediction can be categorized as model-based [19] and model-free approaches (i.e., artificial intelligence methods such as neural-network) [20]. Both linear and non-linear regression techniques can be used to map EMG signals to motion parameters [21,22,23,24]. In this regard, fuzzy neural network approaches are good at mapping both linear and non-linear relationships between inputs and outputs. In this paper, a fuzzy neural network technique is used to establish the mapping of the EMG signal to 3-D of hand motion.

**Figure 1:**
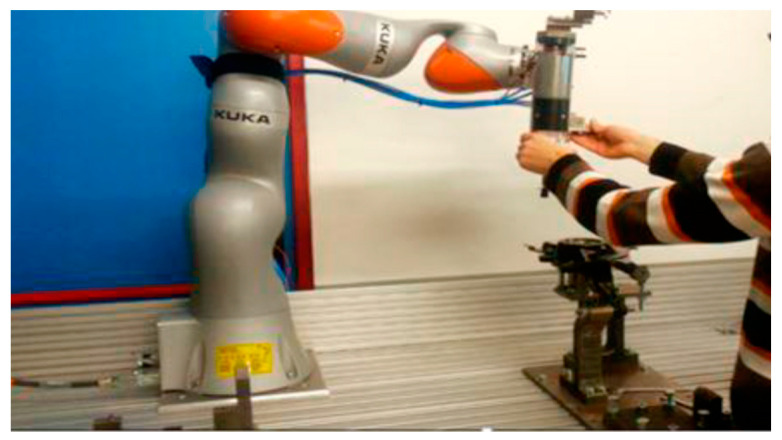
Human–robot collaboration system based on hand motion [25].

Most of the simultaneous and proportional motion prediction studies focused on myoelectric control. The myoelectric control aims at wearable assistive and rehabilitation technologies such as exoskeleton and prosthetics. These applications usually aim to assist users, who lost upper limb functionality, with the consideration of different joints that bring the hand in a certain position. Hence, to regain the upper limb motion functionality, it may be required to consider the simultaneous activation of multiple DOFs. However, in other human–robot collaboration systems, such as the manipulation of a robot in intelligent manufacturing [25] or skill transfer [26,27], the hands alone can be in direct contact with the robot. Figure 1 illustrates an example of the application of human–robot collaboration in manufacturing based on human hand trajectory prediction.

In these collaboration applications, healthy subjects are engaged with a robot partner, and the point of contact between the robot and human alone can be a concern. For instance, in skill transfer, human demonstrations are often simply treated as trajectories, and the robot can mimic the behaviors as demonstrated. The EMG signal is used to estimate the human arm endpoint position, without the concern of the motion of multiple arm joints, that fed to the robot to follow the trajectories.

EMG-based hand motor intention prediction, which aims for information transfer from human to robot, enables the robot to learn and acquire manipulation skills in a complex and dynamic environment [28]. In this application, the prediction of the human arm endpoint position (i.e., the contact point between the hand and robot) alone can establish efficient communication between the robot and human partners. In this regard, Artemiadis et al. [29,30] proposed a state-space model that was used to enable the user to control an anthropomorphic robot arm trajectory in 3-D space. However, in their model, instead of directly estimating 3-D hand position from EMG signal, they used a model that took the joint angles (i.e., estimated angles from EMG signals) and lengths of upper limb as inputs. Even though the coordination of multiple DOFs brings the hand to be at a certain position in space [31], it is not important to consider in our target of application. In our study, instead of first predicting multiple DOFs and then estimating the hand position, we directly estimate the 3-D hand position from the EMG signal. Our approach is similar to the study of Vogel et al. [32] who proposed a method to estimate the position of the hand in space for EMG-based robotic arm systems. However, more complex trajectories, which are different from a point-to-point trajectory such as picking up and putting down objects, were considered in our study.

To the best of our knowledge, there are no studies which consider complex trajectories and directly predict the 3-D hand position from the EMG signal alone. In this paper, we explore how to predict the 3-D hand position for complex trajectories of hand movement directly from the EMG signals alone. We aimed an application of human–robot collaboration where the objects are held by the robot end-effector, while the human controls the robot either remotely by teleoperation or by holding the robot handle. The contribution of this research work is: First, the prediction of a 3-D hand position directly from the EMG signal for complex trajectories of hand movement is studied. The 3-D hand position is predicted from the EMG signal, without the direct consideration of joint movements. Second, our model is constructed for the prediction of a 3-D hand position from the EMG signal. For the efficient communication of a human and robot, the estimation of human intention at the current time might not be adequate and, hence, the future time intention should be estimated. Third, the effects of slow and fast motions on the accuracy of the prediction model are analyzed. Overall, this study proposed a model to predict human motor intention that can be used as information from human to robot. This paper is organized as follows: Section 2 presents the proposed method for the acquisition of the EMG signals and the approaches of intention prediction. Section 3 reports the results and discussion; Section 4 describes the conclusion of the paper.

## 2. Materials and Methods

### 2.1. System Overview

**Figure 2:**
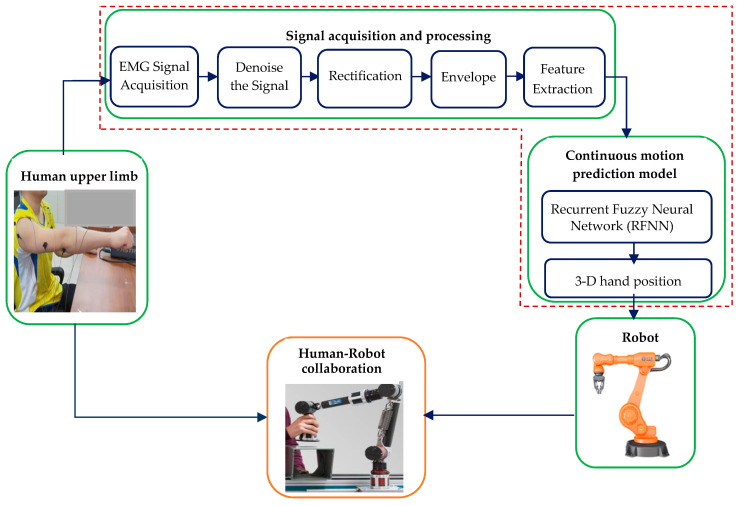
Electromyography (EMG)-based intention prediction of continuous human upper limb motion for human–robot collaboration systems.

As shown in Figure 2, the human–robot collaboration system that is based on the prediction of continuous motion from EMG signals consists of four main elements: Human upper limb, EMG signal acquisition and processing, prediction model, and the robot. First, the EMG signals are collected, while the user performs a complex upper limb motion. Then, the collected EMG signal is preprocessed, and features are extracted from it. A prediction model is used to map the 3-D hand position signal to the extracted features. Finally, the hand position is fed to the controller of the robot to collaborate with the human in shared tasks.

#### 2.1.1. The Human Upper Limb

It consists of several muscles that control the movements of the hand. The human upper limb movement requires the coordination of shoulder, elbow, wrist, and finger joints to perform a set of activities of daily life. In this paper, muscles that are mainly associated with the desired types of movement were identified.

#### 2.1.2. EMG Signal Acquisition and Processing

It involves the selection of muscle position, preparation of the skin, signal acquisition, preprocessing, and feature extraction. First, the acquired raw EMG signals are filtered and rectified. Then, the linear envelope of the signal is generated, and features are extracted.

#### 2.1.3. Continuous Motion Prediction Model

Dynamic model, musculoskeletal model, and artificial intelligence approaches are some of the techniques used to predict continuous motion parameters from EMG signals. In this paper, a recurrent fuzzy neural network is constructed to map EMG signals to 3-D hand positions.

#### 2.1.4. Robot Controller

This part consists of the mechanism to convert the input signal to the robot output signal along with its feedback mechanism. This study aims to propose a method to predict human intention that can be fed to a robot controller to enhance human–robot collaboration. Once the hand position is predicted from the EMG signals, it is fed to the robot control to drive its end effector that holds an object to the desired trajectory. However, the control part is not studied in this paper, as we focused on the offline analysis.

### 2.2. Experimental Protocol

Six right-handed, able-bodied young adults participated in the experiment of hand movement for the desired tasks (age: 20 to 26 years old). The study adhered to the principles of the 2013 Declaration of Helsinki. The online experimental protocol was reviewed and approved by the local research ethics committee, and subjects signed the informed consent forms.

**Figure 3:**
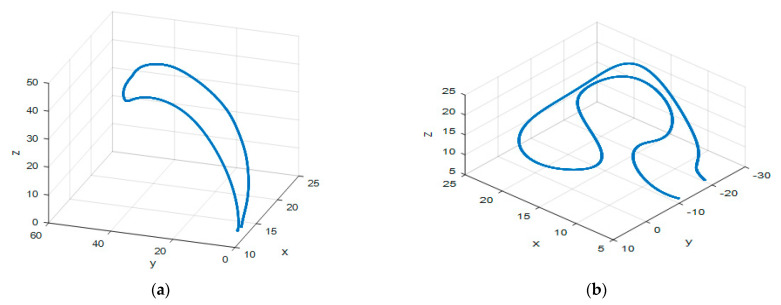
3-D hand trajectories for the designed types of tasks that were drawn from the sample of the experiment. (**a**) Pick up a bottle from the table and pour it into a cup (Task_1); (**b**) manipulation tasks with multiple obstacles (Task_2).

Two different complex tasks associated with a daily activity were designed in 3-D spaces. These motions are (1) picking up a bottle from the table and pouring it into a cup within the range of 10 × 45 × 50 cm (Task_1), and (2) manipulation tasks with multiple obstacles within the range of 20 × 30 × 20 cm (Task_2), as shown in Figure 3. These two motions represent the complex trajectory of tasks executed by the collaboration of a human and robot in applications such as intelligent manufacturing.

**Figure 4:**
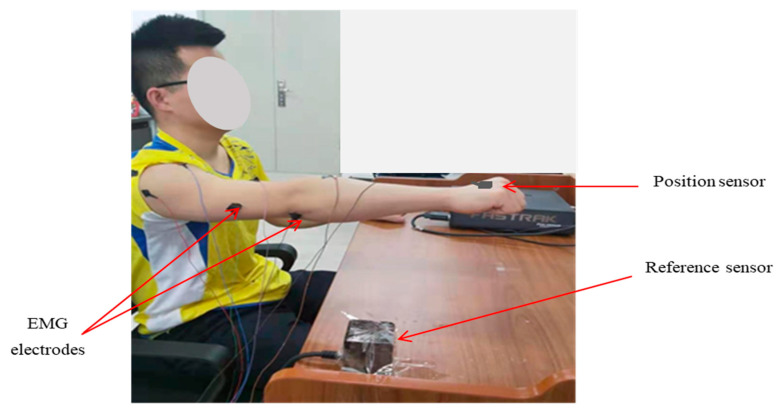
The experimental setup while the subject performs the required task.

After the EMG electrodes and sensors were in place, subjects were instructed to sit with their back straight and in front of a table, where they performed the desired task. At the initial position, the right forearm remained on the table and the shoulder was stabilized in the anatomical reference position with the elbow at 90 degrees flexion. Subjects performed the required movements by grasping their hands. The subjects’ wrist motion was kept fixed throughout the experiment. Figure 4. demonstrates the experimental setup when one of the subjects conducted the required tasks. In the figure, the subjects are seen sitting in front of the table where the required trajectories were performed.

**Figure 5:**
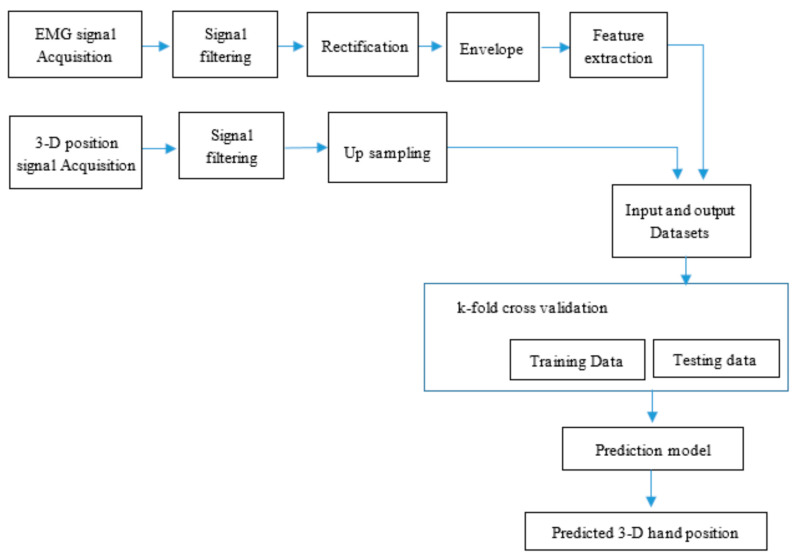
Flow diagram of the proposed method.

The subjects were asked to perform the designed complex trajectories by grasping their hand, while EMG and kinematic data were recorded. Since the objects are held by the robot during the real application, the types of hand grasping is the same for both tasks. The subjects were instructed to move their hands at fast and slow speeds. Since it was challenging to consider several distinct kinds of speed, as the level is determined by the subjects, we limited the consideration of speed for completion of the tasks by fast and slow motion. In this regard, the level of the speed was determined by the subjects based on the advice they received from the authors. To complete the trajectory of each task, fast and slow speed scenarios took approximately 3 and 5 s per cycle, respectively. In one trial, the subjects performed the trajectory 5 times repetitively (i.e., start and finish the task, and back to start point, and so on). In between each cycle, there was a 3-s break. The subjects were encouraged to have adequate rest time if needed. Each trial took about 35 and 45 s for fast and slow speeds, respectively. There were 9 trials for each task and speed; therefore, a total of 36 trials (2 task × 2-speed type × 9 trials) were conducted. During the experiment, both EMG and 3-D hand position signals were collected. After collecting both signals, data processing, construction of prediction model, and analysis of prediction performance were conducted. The flow diagram of the proposed method is shown in Figure 5.

### 2.3. Data Acquisition

**Figure 6:**
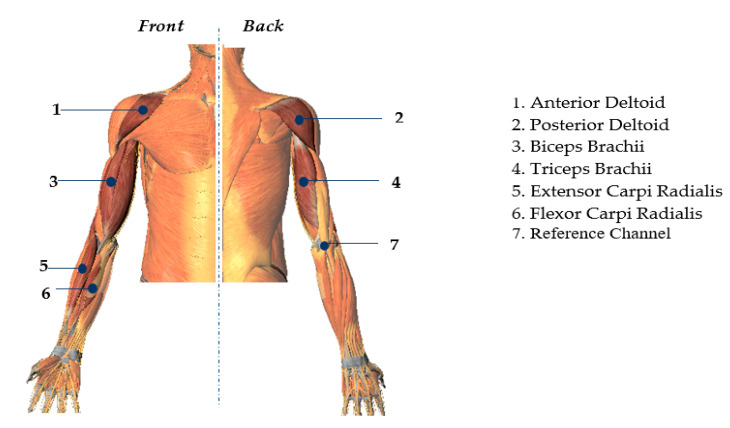
Electrode placement position on upper limb’s muscles. The numbers represent the locations of the muscles.

EMG and Kinematic data were acquired from the subjects simultaneously. Six muscles that predominantly activate joints associated with the desired movements are selected to collect the EMG signals, i.e., anterior deltoid, posterior delhtoid, biceps brachii, triceps brachii, extensor carpi radialis, and flexor carpi radialis. The 7th channel was placed at a bone near to elbow’s joint for reference. A device by Beijing Symtop Instruments Company Limited was used, to collect the EMG signals at the sample rate of 1000 Hz. The skin preparation, placement, and fixation of the electrode were done according to the guideline of SENIAM [10]. The skin, where the electrode was fixed, was cleaned with alcohol to reduce the resistance between the skin and the electrodes. The muscle positions of each EMG electrode are shown in Figure 6.

To collect the kinematic data, we used a FASTRAK tracking system from Polhemus that uses electromagnetic fields to determine the position and orientation of an object. FASTRAK is equipped with position and reference sensors. The important specification of the device is shown in Table 1.

The position sensor was fixed at the hand and the reference sensor was fixed on the table as a reference. The 3-D (x, y, and z Cartesian coordinates) of hand position represent the coordinates of the point in the middle of the opisthenar area (dorsal) of the hand along the three planes (frontal, transverse, and sagittal). The FASTRAK sensor recorded the position of the hand, at a sample rate of 60 Hz in Cartesian space, relative to the reference sensor.

**Figure 7:**
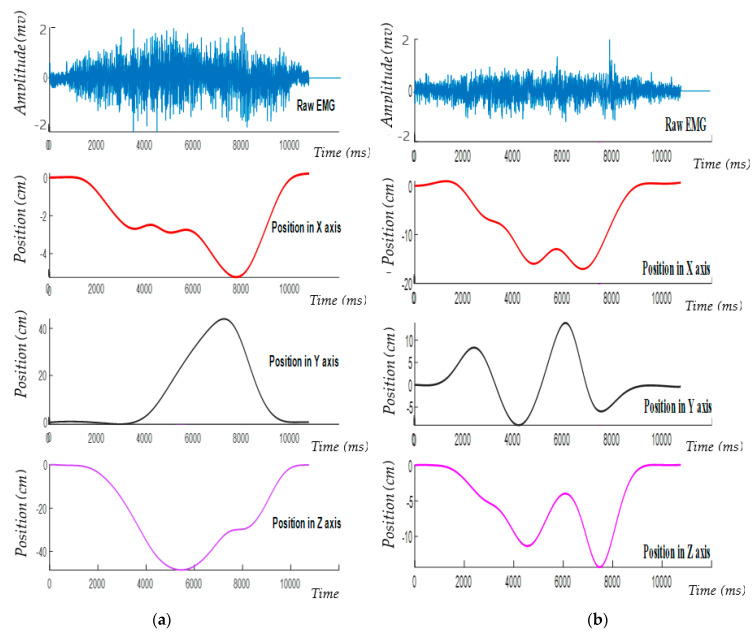
The synchronized EMG and Kinematic signal. (**a**) Task_1; (**b**) Task_2. In both tasks, the top figures represent the amplitude of the raw EMG signal, while the bottom three figures on both the left and right-hand side represent the displacement in the x, y, and z-axis.

The EMG data and kinematics data were synchronized by the NI acquisition device and then sent to the computer as shown in Figure 7. The figure is drawn from the sample of the collected data, while the subjects conducted the desired tasks during the experiment. The figures on the right-hand side (Figure 7a) represent the synchronized EMG and position signal, while the subject performed Task_1. The top figure depicts the amplitude of the EMG signal; whereas the bottom three figures represent the position signal along x, y, and z axes, respectively. Similarly, the figures on the right-hand side (Figure 7b) represent the synchronized EMG and position signal, while the subject performed Task_2. The collected data of EMG and position signals were analyzed on a MATLAB R2010 program.

### 2.4. Data Processing

The collected signals, especially EMG signals, were contaminated with various noises and these noises should be removed from the signals. First, the DC offset was removed from the raw EMG signals by using a Matlab function *detrend*, and the spikes were removed by using median filtering. Then the resulting signals were fully rectified. The fully rectified EMG and position signals were low pass filtered with Butterworth of order 2 and cut off frequency of 2 Hz to generate the linear envelope of EMG signal. The position signal was resampled at a frequency of 1 kHz to be consistent with the sampling frequency of the EMG signals. We checked that there was no significant difference between the original and resampled position signal.

After signal processing, the combinations of root mean square (RMS) and integrated EMG (IEMG) features were extracted. The selection was made after comparisons of the various combinations of seven time-domain features (i.e., mean absolute value, variance, root mean square, waveform length, integrated EMG, slope sign integral, and slope sign change). First, we analyzed the individual performance of mean absolute value, variance, root mean square, waveform length, integrated EMG, slope sign integral, and slope sign change. Then, we analyzed the performance by combining the features until no further improvement of performance was recorded from the combinations of features. As a result, the combination of RMS and IEMG was found to be the best performing features.

The selection of window length can be one of the factors that affect the performance of continuous motion parameter prediction from EMG signals. In this regard, we compared the prediction performance of an overlapping window length of 50 ms, 100 ms, 150 ms, 200 ms, 250 ms, and 300 ms. A total of 12 features (6 channels * 2 feature types = 12 features) were generated for each EMG data point, and they were used as an input for our prediction model.

### 2.5. Prediction Model

**Figure 8:**
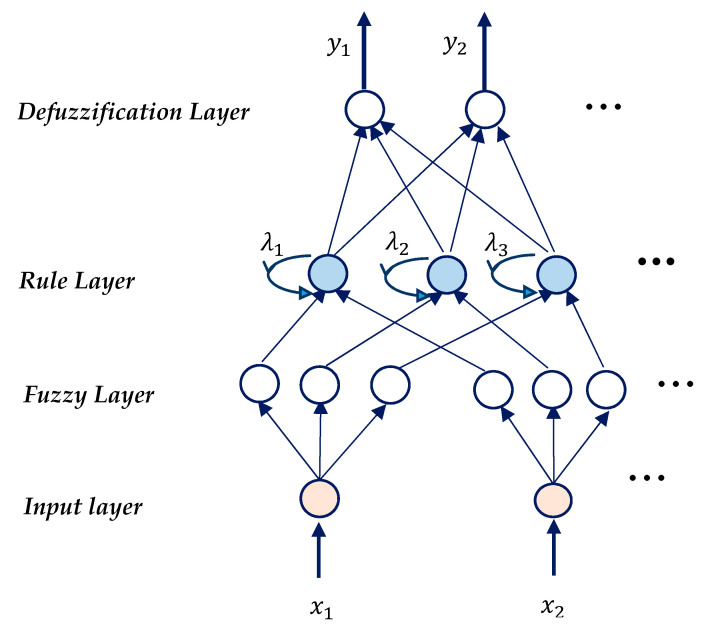
The structure of a recurrent fuzzy neural network. x and y represent input and output vectors, respectively. $\lambda_{i}$ in the rule layer represents the recurrent parameter.

We used a recurrent fuzzy neural network (RFNN) to map the features of EMG signals to the 3-D hand position. The model combines the benefits of both the recurrent structure of the neural network and fuzzy logic. Various forms of RFNN have been used to address time-varying systems [33,34]. Where and how to use the feedback unit in the network differentiates one structure from the other. The structure of the model that is used in this paper is shown in Figure 8.

#### 2.5.1. Structure of RFNN

It has four layers along with a feedback unit in the rule layer. In the subsequent description, $a_{i}{}^{\left(k\right)}$ denotes the $i^{th}$ node output in layer k.

1. Layer 1 (Input layer): It only transmits the input values to the next layer directly; therefore, there is no computation in this layer.

(1) $$a^{\left(1\right)}=x_{i},$$

where, $x_{i}$ is the inputs, i=1,2,…n is number of input variables.

2. Layer 2 (Fuzzification layer): It is defined by the Gaussian membership function that corresponds to the linguistic label of an input variable in layer 1.

(2) $$a^{\left(2\right)}=\exp\left(-\frac{{\left(u_{i}{}^{\left(2\right)}-m_{ij}\right)}^{2}}{\sigma_{ij}{}^{2}}\right),$$

where, $m_{ij}$ is center and $\sigma_{ij}$ is the width of the Gaussian membership function of the $j^{th}$ term of the $i^{th}$ input variable $x_{i}$.

3. Layer 3 (Rule layer): The fuzzy AND operator is used to integrate the fuzzy rules. The output of a rule node $a_{j}{}^{\left(3\right)}$ represents a spatial firing strength of its corresponding rule.

(3) $$a_{j}{}^{\left(3\right)}=\prod_{j}\left(a_{ij}{}^{\left(2\right)}\right),$$

Each node in this layer has a recurrent fuzzy rule node that forms an internal feedback loop.

(4) $$a_{j}{}^{\left(3\right)}=u_{k}^{j}(t)=\lambda_{k}^{j}a_{j}{}^{\left(3\right)}(t)+(1-\lambda_{k}^{j})u_{k}^{j}(t-1),$$

where, $0\leq \lambda_{k}^{j}\leq 1$ is a recurrent parameter that determines the ratio between the contributions of the current and past states.

4. Layer 4 (Defuzzification layer): Each node in this layer is called an output linguistic node and corresponds to one output linguistic variable. This layer performs the defuzzification operation.

(5) $$a_{j}{}^{\left(4\right)}=\frac{\sum_{i}a_{j}{}^{\left(3\right)}w_{ij}}{\sum_{i}a_{j}{}^{\left(3\right)}},$$

where, $w_{ij}$ is the link weight is the center of the membership function of the $i^{th}$ term of the $j^{th}$ output linguistic variable.

#### 2.5.2. Learning of the Model

1. Structure learning: Fuzzy rules are generated from the training data by a clustering algorithm. During learning, a rule is added or reduced based on a firing strength greater than a predefined threshold.

2. Parameter learning: All the parameters (center, width, recurrent parameter, and weight link) are learned by gradient descent algorithm. The cost function E, on the bases of squared error, is defined as

(6) $$E=\frac{1}{2}{\left(y-\hat{y}\right)}^{2},$$

where, y is the desired output and $\hat{y}$ is the actual output.

By using the backpropagation algorithm, the parameters in the corresponding layer are updated

(7) $$m_{ij}(t+1)=m_{ij}(t)-\eta^{m}\frac{\delta E}{\delta m_{ij}},$$

(8) $$\sigma_{ij}(t+1)=\sigma_{ij}(t)-\eta^{\sigma }\frac{\delta E}{\delta \sigma_{ij}},$$

(9) $$\lambda_{ij}(t+1)=\lambda_{ij}(t)-\eta^{\lambda }\frac{\delta E}{\delta \lambda_{ij}},$$

(10) $$w_{ij}(t+1)=w_{ij}(t)-\eta^{w}\frac{\delta E}{\delta w_{ij}},$$

where, $\eta^{m}$, $\eta^{\sigma }$, $\eta^{\lambda }$, and $\eta^{w}$ are the learning rate for the center, width, recurrent parameter, and weight link, respectively. t is the number of iterations.

Processed EMG activity from skeletal muscles precedes mechanical tension by 50–100 ms. This electromechanical delay (the time delay between electromyogram and the related mechanical output) motivated us to develop a prediction model. The features of the EMG signal at the current time t were used to predict the 3-D hand position at the advanced time t+1.

(11) $$\phi (t+1)=\psi (ft_{EMG}(t)),$$

where, ϕ(t+1) is the three-dimensional position of the hand at time t+1, and ψ(.) is a function that takes EMG signal feature $ft_{EMG}(t)$ at time t.

**Figure 9:**
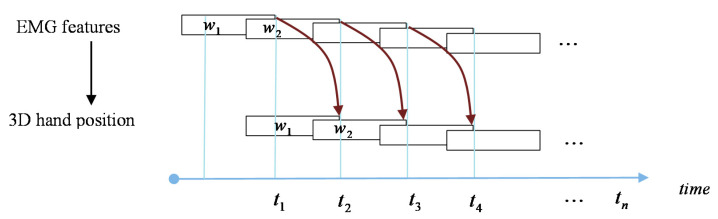
The mapping of EMG features to 3D hand position. The EMG features which are represented with window length $w_{i}$ at time $t_{i}$ are mapped to the 3-D hand position at $t_{i+1}$.

The model in (11) is a prediction model in its strict sense, as it takes the current state of the input to predict the position of the hand at the future time. As shown in Figure 9, the features of the EMG signal at the current time were mapped to the 3D hand position at an advanced time. During data collection, the EMG and kinematics data were synchronized. Subsequently, the signals were processed, and new data points are constructed by time window analysis (i.e., the EMG features are mapped to the 3D hand position). To construct the prediction model in (11), we shifted the position signal to a future time by a window length. In this model, the prediction time horizon of 50–300 ms with an increment of 50 ms was analyzed.

### 2.6. Performance Index

Normalized root mean square error (NRMSE) and Pearson correlation coefficient (CC) were used to measure the prediction accuracy. NRMSE is a widely used performance metric in continuous motion prediction. It is a non-dimensional form of the root mean square error and it is useful to compare root mean square error with different units. Moreover, it can be used to compare models of different scales. CC, which compares the strength of the association between the actual and predicted values [13]. Since NRMSE measures the error while CC measures the similarity between the predicted and actual trajectories, combining the two indexes enables us to comprehensively evaluate the prediction performance.

(12) $$NRMSE=\frac{\sqrt{\frac{1}{n}\sum_{i=1}^{n}{\left(y-\hat{y}\right)}^{2}}}{y_{\max}-y_{\min}},$$

(13) $$CC=\frac{\sum_{i=1}^{n}\left(y-\bar{y}\right)\left(\hat{y}-\bar{\hat{y}}\right)}{\sqrt{\sum_{i=1}^{n}{\left(y-\bar{y}\right)}^{2}\sum_{i=1}^{n}{\left(\hat{y}-\bar{\hat{y}}\right)}^{2}}},$$

where, $\bar{y}$ is the mean of the desired value and $\bar{\hat{y}}$ is the mean value of the actual value. n is the total number of data points, $y_{\max}$ is the maximum of the desired value, and $y_{\min}$ is the minimum of the desired value.

## 3. Results and Discussions

### 3.1. 3-D Prediction Performance for Complex Hand Trajectories

In this study, the proposed model that predict 3-D hand position from EMG signal was validated. We segmented 7 s data from each of the 45 cycles of quick motion, and 9 s data from each of the 45 cycles of slow motion. The segmented data includes the task completion time along with the time of preparation at the beginning of the tasks, and rest at the end of the task (before beginning the second cycle). We used the five-fold cross-validation to train and test our RFNN model. All segmented data of each motion were randomly partitioned into five sets. Each set has 9 samples of the specific task under analysis. The proposed recurrent fuzzy neural network model was trained and tested on the samples from each subject across both tasks. First, the x, y, and z coordinates of the hand position were simultaneously predicted from the features of EMG signals. Then, the prediction performance was measured along with the three coordinates. The quick and slow motions were analyzed independently.

Table 2 presents the mean performance of the subjects across the three coordinates (x, y, and z) in terms of CC and NRMSE. The table presents whenever the tasks are carried out with slow movement. Overall a mean prediction performance of CC = 0.83 and CC = 0.81 are achieved for Task_1 and Task_2, respectively. In terms of NRMSE index, the performance of 0.1179, and 0.1304 are recorded for Task_1 and Task_2, respectively. The CC measures how well the predicted and actual trajectories are similar; and the NRMSE measures the error between them. To comprehend how well the given model was able to predict a given parameter of continuous human intention, it is appropriate to combine two or more performance metrics.

Similarly, Table 3 presents the mean performance of all the subjects across the three coordinates, whenever the tasks are carried out with quick movement. Overall, a mean prediction performance of CC = 0.85 and CC = 0.82 are achieved for Task_1 and Task_2, respectively. While NRMSE is used as a performance index, 0.1051 and 0.1295 are recorded for Task_1 and Task_2, respectively.

As shown in Table 2 and Table 3, the mean accuracy of prediction for Task_1 is greater than Task_2. The hand movement in Task_2 is associated with several obstacles, and it has curved trajectories. However, the difference in the prediction accuracy between the two tasks was insignificant when both NRMSE and CC were used as performance measurements. We statistically analyzed the relationship between the two tasks by using the *p*-value of two-sample t-tests, and we found that the statistical difference between the two tasks for NRMSE (*p* < 0.074) and CC (*p* < 0.216).

**Figure 10:**
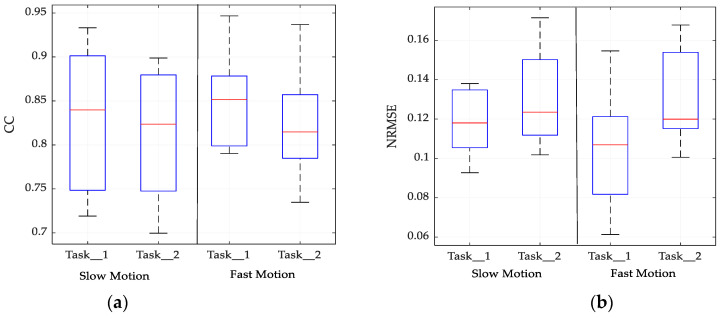
Prediction performance for tasks under consideration. (**a**) Performance in terms of CC index; (**b**) performance in terms of NRMSE index.

Figure 10 presents the mean prediction performance along with the standard error in both metrics. Since EMG signals are subject-specific, the variance in prediction performance across the subjects is observed. The performance of the motion parameter’s prediction from EMG signals can depend on the nature of the task, the model, and the signal processing approaches. In this regard, the accuracy of some tasks could be low because of the complexity of the trajectories of the tasks. It is vital to consider several tasks of daily activity that result in complex trajectories to generalize the relationship between the accuracy of prediction from EMG signals. However, in this particular case, the prediction of a 3-D hand position across the tasks was insignificant.

**Figure 11:**
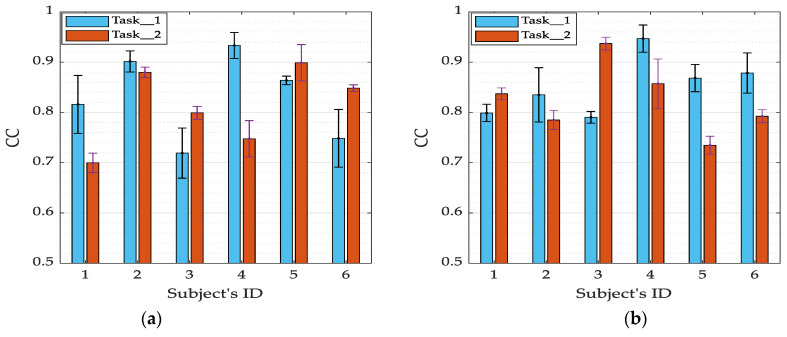
Prediction performance across subjects in terms of CC index. (**a**) Performance for slow-motion; (**b**) performance for quick motion.

Figure 11 presents the performance of intention prediction across each of the subjects. For slow-motion (i.e., Figure 11a), the highest accuracy of CC = 0.93 was found for Task_1 (Subject 4); whereas, for Task_2 the highest accuracy was CC = 0.898 (Subject 5). Similarly, for quick motion (i.e., Figure 11b), the highest accuracy of Task_1 is recorded under Subject 4 (CC = 0.946), while the highest performance for Task_2 is CC = 0.936 (Subject 3). Since EMG signals are subject-specific, the variation of performance across the subjects is as expected. However, for both tasks, the performance across subjects is greater than CC = 0.698 and CC = 0.734 under slow and quick motions, respectively. These results demonstrate that 3-D hand position signal can be predicted, with reasonable accuracy, from EMG signal alone.

Most tasks that are performed by hands in various tasks involve a complex trajectory. The complex movements considered in this study consist of curved trajectories along with multiple obstacles. In hand motion studies of daily activities, the simple motion can be a point to point task which results in approximately straight-line trajectories. Different from complex tasks, hand trajectories formed by point to point tasks are approximately straight. Therefore, the mapping of EMG signals to kinematic parameters of complex trajectories are more challenging than point to point trajectories. The main advantage of the proposed model is no need for consideration for the joint motion to predict the trajectories of the human hand. Therefore, in a real application, the human transfers skills to a robot by mimicking any complex trajectory. The robot predicts the trajectory of the task completion as desired by the human. However, there is a need to improve the performance by considering the challenges of non-stationarity and subject-specific characteristics of EMG signals.

### 3.2. Effects of Speed on Prediction Performance

**Figure 12:**
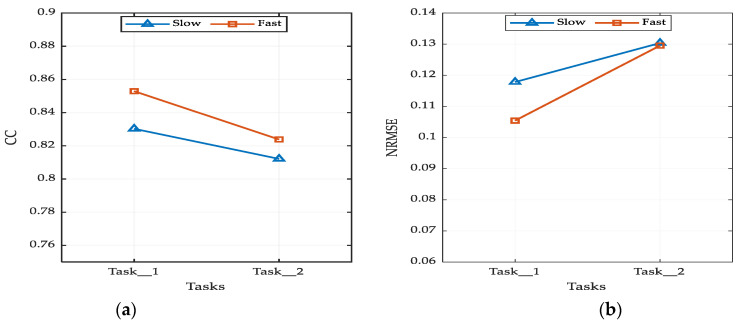
Impacts of speed and tasks on prediction performance. (**a**) Performance in terms of CC index; (**b**) performance in terms of NRMSE index.

Figure 12 shows the impact of speed on the prediction of 3-D hand position from EMG signal; (a) and (b) represent when CC and NRMSE were used as a prediction performance measurement, respectively. Regarding the impact of speed on the accuracy of intention prediction, the lowest NRMSE could be achieved when the tasks were carried out with a fast motion. One of the reasons could be that the trajectory was smooth whenever the speed of the motion was carried out quickly. This situation was evident in Figure 12a, where the CC of both tasks was higher for fast motion.

As shown in Figure 12a, when CC was used as a performance measure, the best prediction performance was obtained for fast motions of Task_1 (R = 0.85), and Task_2 (R = 0.82). Although our approach had the limitation on the strict control of the level of speed (i.e., speed was determined by the subjects), we found that the impact of the speed on the performance of intention prediction was statistically insignificant, Task_1 (*p* < 0.061) and Task_2 (*p* < 0.32). However, the level of impact of speed on the prediction performance can vary across the tasks. For instance, Task_1 was more impacted by the speed of motion than Task_2. The nature of the tasks, such as the dominant plane on which it is carried out and its complexity, can influence the impact of speed.

### 3.3. Impacts of Prediction Time Horizon on Prediction Performance

**Figure 13:**
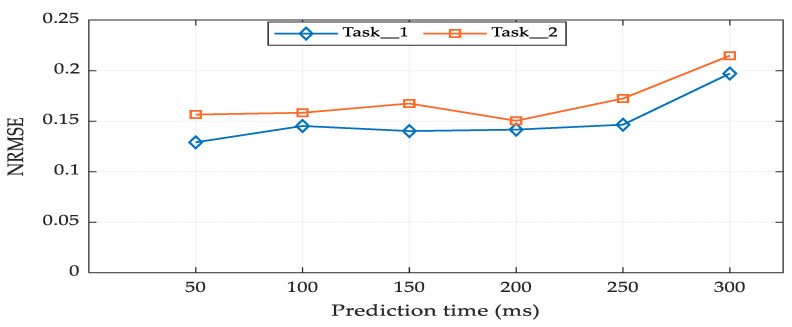
Prediction time horizons and their associated performance.

Figure 13 shows the prediction time horizons and their associated prediction performance. The horizontal line represents the prediction time horizon from 50 to 300 ms, while the vertical line represents the mean performance in terms of NRMSE. The best prediction performance was obtained within 50–200 ms for both tasks. When the prediction time was increased beyond 250 ms, the performance started to decline. Even though EMG signals can be generated about 50–100 ms before the actual motion starts; a delay of computational time is inevitable during a real-time test. We calculated the computational time of our RFNN model by using Matlab R2010a with Intel Core i7-4790 CPU (3.60 GHz) and found that the mean computational time was about 145 ms. Therefore, our result shows that within some range of future time horizon, a 3-D hand position could be predicted well from the EMG signal.

**Figure 14:**
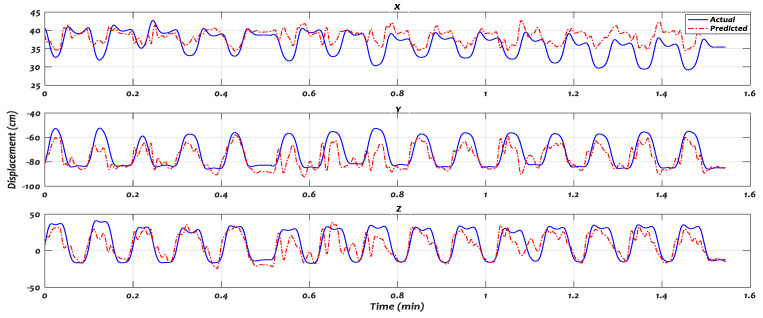
The trajectory predicted by recurrent fuzzy neural network (RFNN) against the actual trajectory of Task 1 in 3-D.

**Figure 15:**
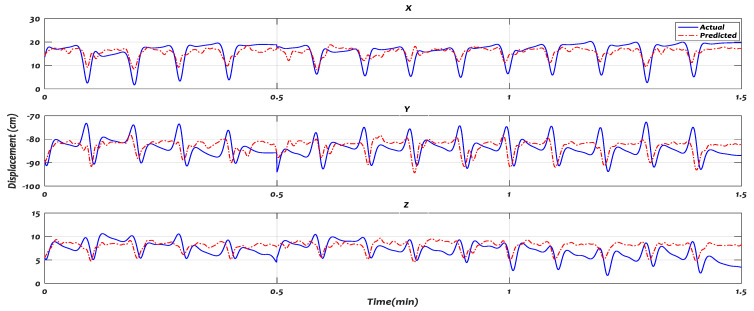
The trajectory predicted by RFNN against the actual trajectory of Task 2 in 3-D.

Figure 14 and Figure 15 illustrate the trajectory predicted by the proposed model against the actual trajectory, when some of the subjects performed Task_1 and Task_2, respectively. The displacements of position x, y, and z coordinates were presented at the top, middle, and down of the figure, respectively. The predicted (red) trajectories were shown alongside the actual (solid-blue line) trajectories.

### 3.4. Comparison with Related Studies

Unlike the classification model [7], where a discrete hand motion is estimated, our study is based on the continuous motion parameter prediction model. Several studies aim to estimate continuous hand motion for rehabilitation or assistive technologies [11,12], however, this study aims where healthy subjects are engaged with a robot to perform a shared task. In assistive technology, the objective is to help the users to gain hand functionality, and the consideration of joint motions are necessary. However, in this study, the aim is to establish communication between a human and a robot with the consideration of a single contact point. Hence, the prediction of 3-D hand position from the EMG signal is used to guide the robot to communicate with a human partner in shared tasks.

In this regard, Artemiadis et al. [29] proposed a state-space model to predict 3-D hand position to control an anthropomorphic robot arm trajectory. However, unlike this study, they first predicted joint angles from EMG signals and then used a model that took the predicted joint angles and lengths of upper limb as inputs. Compared to the work of Xia et al. [21], the prediction performance of our model is equivalent to their recurrent convolutional neural networks model. However, we have used a prediction model in its strict sense as was shown in Equation (11). The model proposed by Vogel et al. [34] estimates the 3-D hand position with high accuracy, however, the trajectory considered in their design is not complex enough in comparison with our designed tasks. Moreover, we analyzed the impact of speed on prediction performance.

This study focuses on the EMG based intention prediction for information transfer from human to robot. In this regard, online testing of the proposed system was not conducted. In addition, only two representative tasks with complex trajectories are considered. These issues are the limitation of our current system, and we will address them in our future studies.

## 4. Conclusions

The prediction of continuous human motion intention is the crucial element of human–robot collaboration systems. EMG Signal is a method used to infer human intention. Several parameters could be predicted from the EMG signal of the human upper limb so that the predicted output is fed to the robot to perform deigned tasks. The position signal is one of these parameters that can be predicted from the EMG signal.

In this study, we aimed to predict 3-D hand position, which can be used for information transfer from human to robot, from EMG signals alone. First, we designed two tasks having complex trajectories and associated with daily life, each with slow and quick motion scenarios. We constructed a prediction model that maps the EMG signals to 3-D hand positions. Then, we analyzed the prediction accuracy of the constructed RFNN model by using CC and NRMSE as performance metrics. Moreover, we analyzed the impact of the speed on the accuracy of prediction. We found that the 3-D hand position can be predicted well within a future time of 250 ms, from the EMG signal alone. Tasks performed with quick motion had slightly a better prediction performance than slow motion. However, the statistical difference in the accuracy of prediction between quick and slow motion was insignificant. The importance of this result is that once the model is trained and the parameters are identified, its performance will not significantly be affected by the change of speeds. Concerning the algorithm, we found that RFNN has a good performance in decoding for time-varying systems. The novelty of this study is we able to predict the 3-D hand position from EMG signal without the consideration of the degree of freedom at joints. The predicted parameter can be directly fed to a robot control to guide the end effector in the required trajectories.

Several limitations that need research attention exist in this study. The first limitation is that several tasks should be designed and analyzed to generalize our results to a general hand motion performed by a person. The second limitation is during EMG signal acquisition, the electrodes could shift from the target muscle or loose skin contact, which can distort the EMG signals and the accuracy of intention prediction. Third, the non-stationary characteristics of EMG signal impair the performance and robustness of motion intention prediction. Generally, EMG has a great potential to predict a continuous human intention, i.e., a 3-D hand position of the upper limb. However, there is a need to improve the performance/accuracy of prediction to accommodate more daily activities that can be used in the human–robot collaboration system. Hence, our future research direction will be to improve the performance of prediction and to test the system in real-time.

## Figures and Tables

**Table 1:** The specification of the FASTRAK 3D motion tracker.

Specifications	Value
Degrees of freedom	6 DOF
Sampling rate	60 Hz
Static accuracy	0.04 cm
Latency	4.0 milliseconds
Operational range	1.52 m

**Table 2:** The performance of intention prediction for slow movement in 3-D (x, y, and z axes).

Tasks	CC				NRMSE			
	CC_x_	CC_y_	CC_z_	Mean	NRMSE_x_	NRMSEy	NRMSEz	Mean
Task_1	0.7513	0.8531	0.8863	0.8302	0.1151	0.1143	0.1242	0.1179
Task_2	0.8156	0.7951	0.8279	0.8129	0.1278	0.1267	0.1366	0.1304

**Table 3:** The performance of intention prediction for quick movement in 3-D (x, y, z).

Tasks	CC				NRMSE			
	CC_x_	CC_y_	CC_z_	Mean	NRMSE_x_	NRMSEy	NRMSEz	Mean
Task_1	0.8524	0.8234	0.8827	0.8529	0.1068	0.1071	0.1014	0.1051
Task_2	0.8296	0.8072	0.8347	0.8238	0.1306	0.1334	0.1246	0.1295

## Data Availability

Data sharing is not applicable to this article.
